# Effectiveness of Anthocyanin-Rich Sour Cherry Extract on Gliadin-Induced Caco-2 Barrier Damage

**DOI:** 10.3390/nu15184022

**Published:** 2023-09-17

**Authors:** Ágnes Klusóczki, Boglárka Oláh, Dominik Hosszú, Ferenc Fenyvesi, Judit Remenyik, Judit Homoki, Alexandra Gyöngyösi, Ildikó Bácskay, Judit Váradi

**Affiliations:** 1 Institute of Healthcare Industry, University of Debrecen, H-4032 Debrecen, Hungary; klusoczki.agnes@euipar.unideb.hu; 2 Department of Pharmaceutical Technology, Faculty of Pharmacy, University of Debrecen, Nagyerdei Körút 98, H-4032 Debrecen, Hungary; bogocaolah@gmail.com (B.O.); hosszudominik56@gmail.com (D.H.); fenyvesi.ferenc@pharm.unideb.hu (F.F.); bacskay.ildiko@pharm.unideb.hu (I.B.); 3 Institute of Food Technology, Faculty of Agricultural and Food Sciences and Environmental Management, University of Debrecen, H-4032 Debrecen, Hungary; remenyik@agr.unideb.hu (J.R.); homoki.judit@agr.unideb.hu (J.H.); 4 Department of Pharmacology, Faculty of Pharmacy, University of Debrecen, H-4032 Debrecen, Hungary; gyongyosi.alexandra@pharm.unideb.hu

**Keywords:** PT-gliadin, anthocyanin, Caco-2, permeability, ROS

## Abstract

Several types of gluten-related disorders are known, in which the common starting point is gluten-induced zonulin release. Zonulin results in varying degrees of increased permeability in certain gluten-related disorders but is largely responsible for the development of further pathogenic processes and symptoms. Therefore, it is important to know the barrier-modulating role of individual nutritional components and to what extent the antioxidant substance supports the protection of gliadin-induced membrane damage with its radical scavenging capacity. We investigated the pH dependence of the gliadin-anthocyanin interaction using UV photometry, during which a concentration-dependent interaction was observed at pH 6.8. The barrier modulatory effect of the anthocyanin-rich sour cherry extract (AC) was analyzed on Caco-2 cell culture with pepsin-trypsin-resistant gliadin (PT-gliadin) exposure by TEER measurement, zonula occludens-1 (ZO-1), and Occludin immunohistochemistry. In addition to the TEER-reducing and TJ-rearranging effects of PT-gliadin, NF-κB activation, an increase in cytokine (TNF-α, IFN-γ, and IL-8) release, and mitochondrial ROS levels were observed. We confirmed the anti-inflammatory, stabilizing, and restoring roles of AC extract during gliadin treatment on the Caco-2 monolayer. The extract was able to significantly reduce cytokine and ROS levels despite the known interaction of the main components of the extract with PT-gliadin.

## 1. Introduction

Celiac disease (CD) is a chronic, T-cell-mediated enteropathy with autoimmune features induced by the consumption of gluten-containing cereals in genetically susceptible people, occurring in 1 out of 100–300 people worldwide [1]. CD is determined by a disease-specific autoantibody response targeting tissue transglutaminase (tTG) [2]. During gluten consumption, IgA-class tTG autoantibodies are circulating in serum and are also deposited in the small bowel mucosa [3]. Gliadin is one of the main components of gluten; it contains large amounts of proline and glutamine, which are only partially digested by human proteases, resulting in large, immunogenic peptides [4]. The undigested gliadin forms several types of peptide fragments, such as pepsin-trypsin-resistant gliadin (PT-gliadin), in the small intestine lumen.

The gliadin peptides provoke a strong adaptive immune response. First, they are deaminated by the tissue-transglutaminase enzyme, and then the deaminated gliadin peptides are recognized by human leukocyte antigen (HLA)-DQ-DQ-8 molecules. The aforementioned molecules are expressed on antigen-presenting cells (APCs), which present the gliadin peptide to CD4^+^ T cells. The activated complex of gluten-CD4^+^ T cells produces high levels of pro-inflammatory cytokines, which subsequently activate the nuclear factor kappa-B (NF-κB) pathway [5,6,7]. In the histological changes of CD, the secretion of TNF-α and IFN-γ is usually dominant among other pro-inflammatory cytokines.

Gastroenterology distinguishes several types of gluten-related disorders (GRD) with intestinal symptoms, such as CD, non-celiac gluten sensitivity (NCGS), and wheat allergy [8]. The most frequently examined and serious problem is gluten-sensitive enteropathy, i.e., celiac disease, but gluten can be identified as an inducer in many other pathologies as well [9,10]. The pathomechanism of these disorders is different compared to the previously highlighted celiac disease; however, the immunogenic peptides 33-mer and 25-AA formed during the digestion of gluten induce an immune response in enterocytes and intestinal immune cells. Gluten-related induction results in zonulin release, a MyD88-dependent mechanism mediated by the CXCR3 receptor [11]. Activation of CXCR3 by gliadin leads to the rearrangement of the TJ protein [12].

Tight junctions (TJs) are believed to be the major junctions responsible for regulating intestinal mucosa barrier permeability and are composed of several types of proteins, including Occludin, claudins, and zonula occludens (ZOs), and controlled by a set of over 50 proteins. Intestinal barrier integrity is regulated by complex interactions between TJ-associated proteins, including ZO-1 and Occludin, the best-studied components of the junctions [13]. TJ proteins are highly selective and responsive, opening or closing as a result of inflammatory or regulatory cytokines or foreign antigens. For example, one of these foreign antigens that has an effect on TJs is gliadin, which is a water-insoluble portion of the gluten protein complex [14]. Previously, it has been shown that gliadin has a disjunctive effect on TJ proteins, namely on actin and ZO-1, which results in increased gut permeability [15].

In these current studies, we investigated the barrier-modulating effect of an anthocyanin-rich sour cherry extract (AC) in a gliadin-induced in vitro model system. To achieve this aim, we utilized the intestinal epithelial cell line Caco-2, which is widely used as a model for in vitro studies of human intestinal epithelium functions [16].

Many studies have introduced the beneficial effects of anthocyanins isolated from Hungarian sour cherries, which are characterized by a high cyanidin glycoside content [16,17]. Several studies have proven that anthocyanin-rich cherry extract (AC) has diverse effects, including anti-inflammatory, antioxidant, and vasoactive effects [18,19,20]. These results indicate that AC could be protective against inflammatory bowel disease and have beneficial effects on irritable bowel syndrome [21]. In this study, the AC has been used as a model of biologically active nutrients.

In our latest publication, we investigated the modulating effect of anthocyanin-rich extract (AC) on TJ proteins in inflammatory Caco-2 and HUVEC monolayers. We proved the positive effect of AC, which affected the expression of TJs both at protein and mRNA levels.

The aim of the current study was to investigate the intestinal barrier integrity and defensive effect of anthocyanin extract on gliadin-induced inflammation in Caco-2 cells. The binding of gliadin to anthocyanin has already been confirmed in the case of different anthocyanin extracts [22,23,24]. However, the pH dependence of the interaction, which can be modified under the different pH conditions of the digestive system, has not yet been investigated. The medium with a pH of 6.8 is typical for the upper part of the small intestine, where even more significant anthocyanin utilization is possible, and is close to the pH of cell culture experiments. However, the degree of gliadin-anthocyanin interaction has not yet been investigated in this pH range, so we considered it important to have a background knowledge of the physiological effects of anthocyanins.

## 2. Materials and Methods

### 2.1. Materials

Gliadin from wheat, pepsin, and trypsin were purchased from Sigma-Aldrich (Budapest, Hungary). Sour cherry anthocyanin extract (AC) was prepared by the Department of Feed and Food Biotechnology, University of Debrecen (Hungary), as described previously [16]. The main anthocyanin components in the extract were cyanidin-3-O-glucosyl-rutinoside, cyanidin-3-O-rutinoside, and cyanidin-3-O-monoglucoside. The anthocyanin components of the AC extract in the ‘Újfehértói fürtös’ variety were determined by UHPLC. Based on the molar ratio of the anthocyanin components, we calculated the average molar mass of AC, for which we obtained a value of 574.5 g/mol [17]. This value was used to calculate the μM concentrations in experiments. All other reagents were ordered from Sigma-Aldrich (Budapest, Hungary).

### 2.2. Preparation of Pepsin-Trypsin-Resistant Gliadin (PT-Gliadin)

PT-gliadin was prepared as follows: 10 g of gliadin was dissolved in 100 mL of 0.2 mM HCl and incubated with 250 mg of pepsin for 4 h at 37 °C. Then, 1 mM NaOH was used to adjust the pH of the solution to 8.0. After the pepsin digestion, the product was further digested by 250 mg of trypsin for an additional 4 h in an oscillator at 37 °C, shaken at 50 rpm. Furthermore, the mixture was boiled for 30 min to inactivate the enzymes before lyophilizing the solution and then storing it at −20 °C. For each experiment, PT-gliadin was suspended in PBS at a final concentration of 1 mg/mL.

### 2.3. Cell Culturing for Permeability Studies

The human Caco-2 intestinal epithelial cell line was maintained in DMEM culture medium supplemented with 10% fetal bovine serum (FBS) in an incubator with 5% CO_2_ at 37 °C. A transwell system with 12 inserts was used for the experiments, which were permeable polycarbonate filters with a 0.4 µm pore size. Caco-2 cells were seeded in the apical (upper) chamber at a high density (2 × 10^5^ cells/chamber) with 500 µL of the medium. The basal (lower) chamber contained 1500 µL of medium, which was changed every third day.

### 2.4. Real-Time Monitoring of Cell Index (RTCA)

The XCELLigence system was developed to allow label-free, dynamic monitoring of cell proliferation and viability in real-time. Furthermore, 2 × 10^4^ cells per well were seeded into 100 µL of media in E-plate 16 in duplicates and maintained at 37 °C with 5% CO_2_. E-plates are coated with gold sensor arrays to measure electrical impedance. Approximately 24 h after seeding, when the cells were in the log growth phase, they were treated with 100 µL of media containing gliadin/gliadin + AC. The concentration range (0.1–10 mg/ml) of PT-gliadin was determined based on the viability test previously performed on Caco-2 cells (see Figure S1). Untreated media was used as a control. The proliferation of the cells was monitored every 30 min using the xCELLigence system for 72 h. The results were converted to the cell index by software.

### 2.5. UV Spectroscopy and Determination of AC Adsorption by PT-Gliadin

AC extract was dissolved, and 50–500 μM solutions were prepared in 6.8 pH water and 3.0 pH citrate buffer. The spectra of the pH 6.8 and pH 3.0 AC solutions were recorded, and the absorbance (A_0_) at the absorption maximum (515 nm) was measured. Afterwards, 1 mg of gliadin was added and mixed with the AC solutions, which were then incubated at 37 °C for 60 min. The solutions were centrifuged for 5 min at 1000× *g*, and then the absorbance of the supernatants was measured (A_60_). The A_60_-A_0_ values were calculated and graphed by GraphPad Prism 8.0 software (GraphPad Software Inc., La Jolla, CA, USA). The adsorbed AC ratio was expressed in % according to the following:

AC_adsorbed%_ = (C_AC post_/C_AC pre_) × 100 (1)

C_AC post_: concentration of AC in μM after incubation with PT-gliadin.

C_AC pre_: concentration of AC in μM before incubation with PT-gliadin.

### 2.6. Transepithelial Electrical Resistance (TEER) Measurements

The determination of transepithelial electrical resistance provides information about the uniformity of the Caco-2 cell layer on the filter support and the integrity of tight junctions (TJs) formed between the polarized cells. Caco-2 cells were seeded at a density of 2 × 10^5^ to insert into a 12-well plate. The electrode was immersed in the upper and basal medium chambers, and resistance was measured by a portable volt-ohmmeter like the Millicell-ERS Voltmeter. Caco-2 monolayers were treated with the test substances as follows: First group: untreated control; second group: 1 mg/mL gliadin treatment; third group: 100 µM anthocyanin (AC) treatment; fourth group: 1 mg/mL gliadin + 100 µM AC combined treatment. Cells were treated for 3 days (72 h), every day at the same time. TEER values were monitored every 24 h; time zero was considered a TEER value of 100%.

### 2.7. Immunofluorescence Staining of ZO-1 and Occludin

Morphological changes were investigated by immunofluorescence of zonula occludens-1 (ZO-1) and Occludin plasma membrane proteins. Cells were seeded onto sterile glass coverslips placed into 12-well plates at a density of 50,000 cells per slide. The experiment was designed as follows: Caco-2 cells were divided into four groups. The first group was untreated cells, which served as a negative control. The second group of cells was stimulated with 1 mg/mL gliadin for 24 h. The third group was treated with 100 µM AC. In the fourth group, combined treatment was applied: 1 mg/mL gliadin and 100 µM AC. Cells were then washed with PBS and fixed with ice-cold methanol-acetone (50–50%) for 10 min. After a washing step, cells were blocked with FBS for 30 min. As a next step, anti-ZO1 (Cat.No. 40-2200, Invitrogen, Waltham, MA, USA) and rabbit Occludin primary antibodies (Cat.No. 71-1500, Invitrogen) were added to the cells for 1 h. Next, Alexa Fluor-488 anti-rabbit secondary antibodies (Cat.No. A32731, Invitrogen) lasted for 1 h. Bis-benzimide dye (Hoechst 33342) was used to stain cell nuclei. Microscopic evaluation was performed using a Zeiss Axioscope A1 fluorescence microscope (HBO 100 lamp) (Carl Zeiss Microimaging GmbH, Göttingen, Germany). Images were analyzed with ZEN 2012 v.1.1.0.0. software (Carl Zeiss Microscopy GmbH).

### 2.8. Permeability Study

Caco-2 cells were seeded at a density of 2 × 10^5^ cells on inserts and differentiated until confluence. After reaching confluence, the TEER of each cell layer was determined before the treatments. Wells showing a TEER value over 900 Ω cm^2^ were only considered for the experiment. Then, cells were treated with PT-gliadin, AC, or a combination cocktail of PT-gliadin and AC in cell culture medium. Plates were incubated at 37 °C in an incubator with 5% CO_2_ for 24 h. Moreover, Caco-2 cell layers were washed twice with Hanks’ Balanced Salt solution (HBSS) and incubated for 20 min at 37 °C. As a next step, 50 µg/mL Lucifer Yellow (LY) was dissolved in HBSS in the upper compartment. The permeation study was conducted at 37 °C for 2 h in an incubator. The incubation samples were collected from the lower compartment at 30, 60, 90, and 120 min. The withdrawn volume was replenished by HBSS. The fluorescence intensity of permeated LY was determined photometrically at an excitation wavelength of 450 nm and an emission wavelength of 520 nm with a fluorescence multiwall plate reader (Fluostar Optima, BMG Labtechnologies, Ortenberg, Germany). The apparent permeability coefficients for LY were calculated as described in the following equation:

P_app_ = dQ/dt × 1/(C_0_ × A) (2)

where P_app_ is the apparent permeability coefficient (cm/s); dQ/dt is the permeability rate of substances (mol/s); C_0_ is the initial concentration of the substances in the upper compartment (mol/mL); and A is the surface area of the membrane (cm^2^).

### 2.9. NF-κB Pathway Activation Study

The NF-κB pathway activation in Caco-2 cells was investigated via p65 nuclear immunofluorescence staining. Briefly, cells (50,000 cells/slide) were seeded on sterile glass coverslips, placed into 12-well plates, and cultured in DMEM. When the cells reached the appropriate confluence, the media was renewed, and the cells were incubated with 1 mg/mL gliadin to induce inflammation for 1 h at 37 °C. Then, cells were washed twice with HBSS and fixed with ice-cold methanol-acetone (50–50%) for 10 min, and non-specific binding sides were blocked with fetal bovine serum (FBS) for 15 min. After this, cells were washed with HBSS and incubated with 2 µg/mL of primary anti-p65 polyclonal antibody (Cat.No. sc-8008 AF488, Santa Cruz Biotechnology, Dallas, TX, USA) for 1 h at 37 °C, followed by washing with Hanks Balanced Salt Solution (HBSS) three times. Next, 5 µg/mL of secondary antibody (Alexa Fluor 488 goat anti-rabbit) was applied for 1 h at 37 °C in the dark. Bis-benzimide dye (Cat. No. Hoechst 33342) was used to stain cell nuclei for 10 min at 37 °C. After this, cells were washed once with HBSS, and the glass coverslips were glued to microscope slides. Fluorescence microscopy measurements were carried out by a Zeiss Axioscope A1 (Carl Zeiss AG, Jena, Germany) fluorescent microscope. Images were analyzed using ZEN 2012.v.1.1.0.0. software (Carl Zeiss Microscopy GmbH, Göttingen, Germany).

### 2.10. Measurement of TNF-α, IFN-γ, and IL-8 Production with the ELISA Method

A transwell system with 12 inserts was used for the experiments, which contained polycarbonate filters with 0.4 μM pore sizes. Caco-2 cells were seeded in the apical (upper) chamber at a density of 2 × 10^5^ cells/chamber. The basal (lower) chamber contained 1 mL of medium. When the cells fully grow the membrane, the culture medium is removed, and the cells are incubated for 24 h with the following: 100 μM anthocyanin (AC), 1 mg/mL PT-gliadin (G), and combined treatment with AC and PT-gliadin (AC + G). Untreated culture medium served as a control. Then, supernatants from the apical chamber were collected, and human ELISA kits were used to measure the cytokine production. TNF-α (Cat.No. BMS223-4, Invitrogen), IFN-γ (Cat.No. KHC4021, Invitrogen), and IL-8 (Cat.No. BMS204-3, Invitrogen) were investigated by the supernatant according to the manufacturer’s instructions.

### 2.11. Assessment of Mitochondrial ROS

Caco-2 cells were seeded on a 24-well plate at 1 × 10^4^ cells/well density. The next day, cells were treated with 1 mg/mL gliadin, 100 μM AC, and the combination cocktail of gliadin and AC. Untreated cells served as negative controls. After 24 h of treatment, the medium was removed, and cells were washed three times with HBSS. MitoSOX Red (Cat.No. M36008, Invitrogen) was added to the cells in a 1 μM concentration for 10 min at 37 °C in the incubator. Furthermore, the dye was removed, and cells were washed three times with HBSS. After washing, the trypsin-EDTA solution was added to the wells for 10 min. Then, media was added to stop trypsin. Cells were collected and centrifuged at 1100 rpm for 6 min. As a next step, the supernatant was removed, and cells were suspended in 300 μL/well HBSS for flow cytometric investigation.

### 2.12. Statistical Analysis

The statistical analyses were performed by GraphPad Prism 8.0 software (GraphPad Software Inc., La Jolla, CA, USA). Data are presented as means ± SD, number of replicates *n* = 3, *n* = 6 (in Method 2.4.), and *n* = 10 (in Method 2.9.), and given in every figure captured. Comparisons of groups were performed using one-way and two-way ANOVA and Bonferroni multiple comparison tests. Differences were considered not significant at *p* > 0.05.

## 3. Results

### 3.1. Real-Time Monitoring of Cell Index

**Figure 1:**
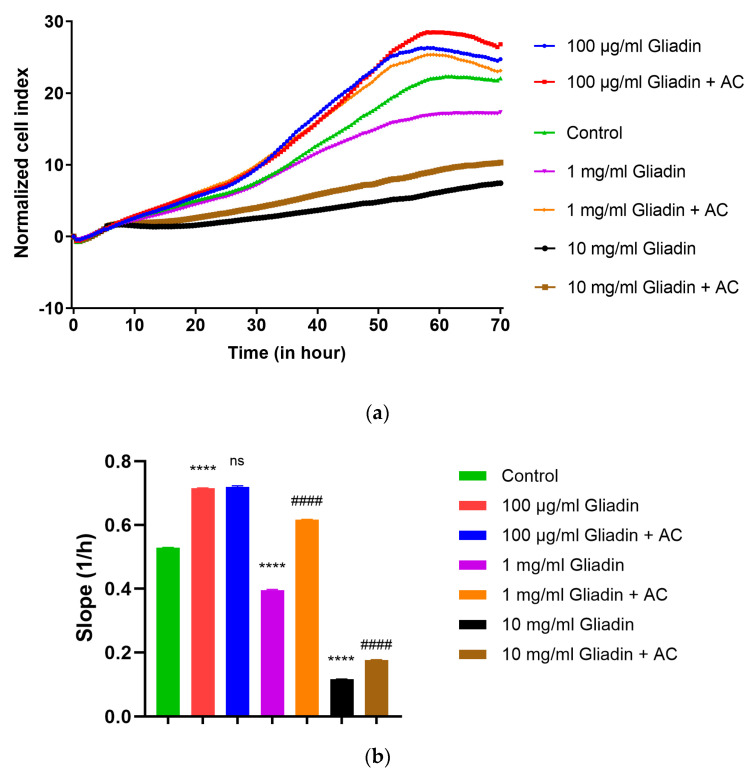
Impedance measurements with real-time cell analysis (RTCA). The kinetics of endothelial cell response to treatment were monitored (**a**), and the curve slopes were calculated by XCeLLigence software (**b**). Caco-2 monolayers were treated with different concentrations of gliadin (100 μg, 1 mg, and 10 mg/mL) in the presence or absence of 100 μM anthocyanin (AC). Cell indexes were monitored every 30 min. Data are presented as means ± SDs, *n* = 6. Differences were considered significant at *p* < 0.05; **** (C vs. G) *p* < 0.0001, #### (G vs. AC + G) *p* < 0.0001, and ns means not significant compared to the control.

The proliferation effect of different gliadin concentrations on Caco-2 cells was investigated by the RTCA method (Figure 1). Furthermore, 100 μg/mL gliadin and combined AC treatment resulted in a significantly proliferative effect compared to untreated cells, and the efficacy of AC treatment was insufficient compared to gliadin treatment. Treatment with 10 mg/mL gliadin showed a significantly reduced proliferation effect, which already indicates toxicity. Treatment with 1 mg/mL gliadin significantly inhibited the proliferation of Caco-2 cells, and AC treatment significantly improved the cell index to the level of the control group. Thus, we used a 1 mg/mL gliadin treatment in our further experiments.

### 3.2. Investigation of the AC Adsorption of PT-Gliadin

**Figure 2:**
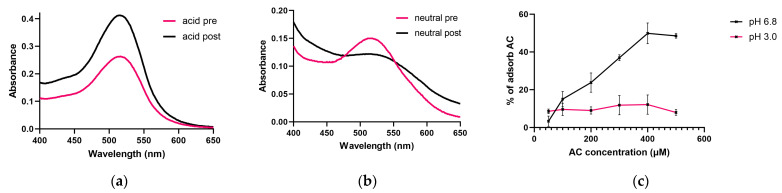
Investigation of AC binding by PT-gliadin. Representative spectrum of AC in pH 3.0 aqueous solution. Acid pre means before, and acid post means after treatment with PT-gliadin (**a**). Representative spectrum of AC in pH 6.8 aqueous solution. Neutral pre means before, and neutral post means after treatment with PT-gliadin (**b**). Investigation of pH and concentration dependence of AC binding of PT-gliadin, λ = 515 nm (**c**). Data are presented as means ± SD, *n* = 3.

The degree of interaction was tested at 3.0 and 6.8 pH, and the spectrum was recorded before and after the gliadin treatment on both pHs. In the spectra recorded in the pH 3.0 medium, we did not observe the shift of the absorption maximum, which assumes the absence of interaction. The absorption maximum was measured between 512 and 515 nm before and after treatment (Figure 2a). However, in the neutral medium, at pH 6.8, we experienced a smaller bathochrome shift from 513–516 nm to 522–524 nm (Figure 2b).

The degree of absorption was determined with increasing AC content at 515 nm. According to our results, we did not experience a significant difference in AC binding in an acidic medium; nearly 10% were bound to PT-gliadin (Figure 2c). However, in a neutral medium, the degree of adsorption increased almost linearly by increasing the concentration of AC; only at the highest concentration (500 μM AC) was there an unchanged degree of binding, which may indicate saturation. During our experiments, we used 100 μM AC in addition to 1 mg/mL PT-gliadin, at which ratio the AC adsorption of gliadin shows a value of 18%.

### 3.3. Measurement of Transepithelial Electric Resistance (TEER)

**Figure 3:**
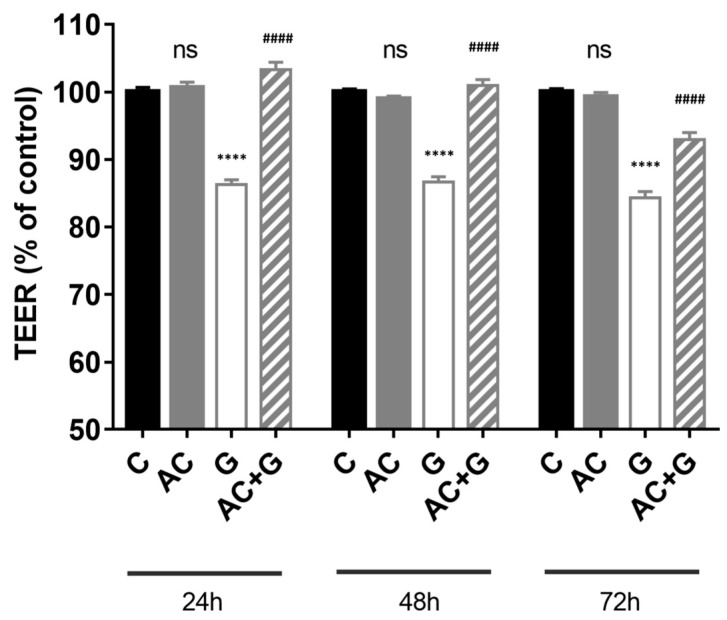
The barrier function of the Caco-2 cell monolayer was investigated by transepithelial electric resistance (TEER). The cells were treated with 1 mg/mL gliadin, 100 µM AC, and a combined treatment of 1 mg/mL gliadin and 100 µM AC for three days. Treatments were performed every 24 h. TEER values are shown as percentages of the untreated controls. Data are presented as means ± SDs, *n* = 3. Differences were considered significant at *p* < 0.05; **** (C vs. G) *p* < 0.0001, #### (G vs. AC + G) *p* < 0.0001, and ns means not significant.

Caco-2 cells were seeded and grown on Corning Transwell polycarbonate filters until the TEER value reached 900 Ω cm^2^. The monolayers were treated with 1 mg/mL gliadin, 100 μM AC, or a combination of both for 72 h (treatment every 24 h for 3 days). Differentiated Caco-2 monolayers expressed high TEER, indicating a tight paracellular barrier. Gliadin treatment reduced the resistance of Caco-2 monolayers after 72 h compared to the TEER changes of the control, untreated group (84.33 ± 0.92%), presented in Figure 3. AC treatment alone did not result in a significant difference in TEER values (99.23 ± 0.69%). Furthermore, treatment with a combination of AC and gliadin resulted in a higher TEER value compared to the group treated with gliadin (93 ± 1.00%).

### 3.4. Immunohistochemical Staining of TJ Proteins

**Figure 4:**
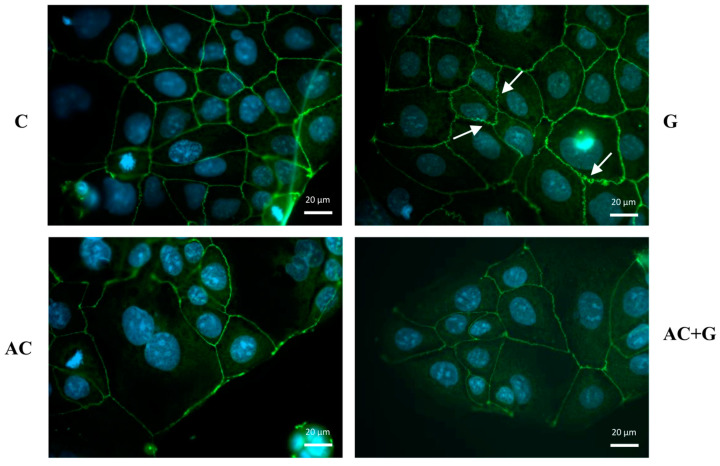
Effects of anthocyanin (AC) and/or PT-gliadin treatment on zonula occludens-1 (ZO-1) morphology of Caco-2 cells. The white arrows show the ZO-1 protein rearrangement that occurs under the influence of PT-gliadin. Cells were treated for 24 h with culture medium (C), 1 mg/mL PT-gliadin (G), 100 µM AC, or a combination of 100 µM AC (pretreatment) and 1 mg/mL PT-gliadin (AC + G). Green color: immunostaining of ZO-1; blue color: staining of cell nuclei. The scale bar is 20 µm.

**Figure 5:**
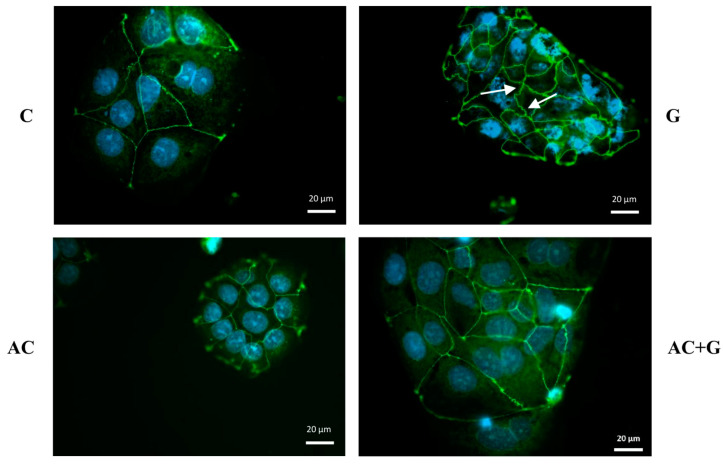
Immunofluorescence staining of Occludin. Effects of anthocyanin (AC) and/or PT-gliadin (G) treatment on junctional morphology of Caco-2 cells. The white arrows show the Occludin protein rearrangement that occurs under the influence of PT-gliadin. Cells were treated for 24 h with culture medium (C), 1 mg/mL gliadin (G), 100 µM AC or a combination of 100 µM AC (pretreatment) and 1 mg/mL PT-gliadin (AC + G). Green color: immunostaining for Occludin; blue color: staining of cell nuclei. The scale bar is 20 µm.

The tight paracellular barrier of Caco-2 cells is indicated by the belt-like, continuous appearance of ZO-1 (see Figure 4) and Occludin (see Figure 5) proteins, which are clearly visible in the monolayers of the control group. In the cells treated with gliadin, the staining pattern shows the cytoplasmic redistribution of junctional proteins; a zig-zag pattern was observed in the monolayer, with membrane fragments lacing off in some places and uneven junctional staining.

The arrangement of ZO-1 and Occludin proteins in epithelial cells treated with anthocyanin extract (A) was like that of the control group; the continuity and belt-like structure of TJ proteins can be seen on the non-marginal cells. In the case of gliadin induction, treatment by A moderated the rearrangement of cell-to-cell connections, which is confirmed by the uniform staining and continuous belt-like appearance of ZO-1 and Occludin.

### 3.5. NF-κB Activation Pathway Study

**Figure 6:**
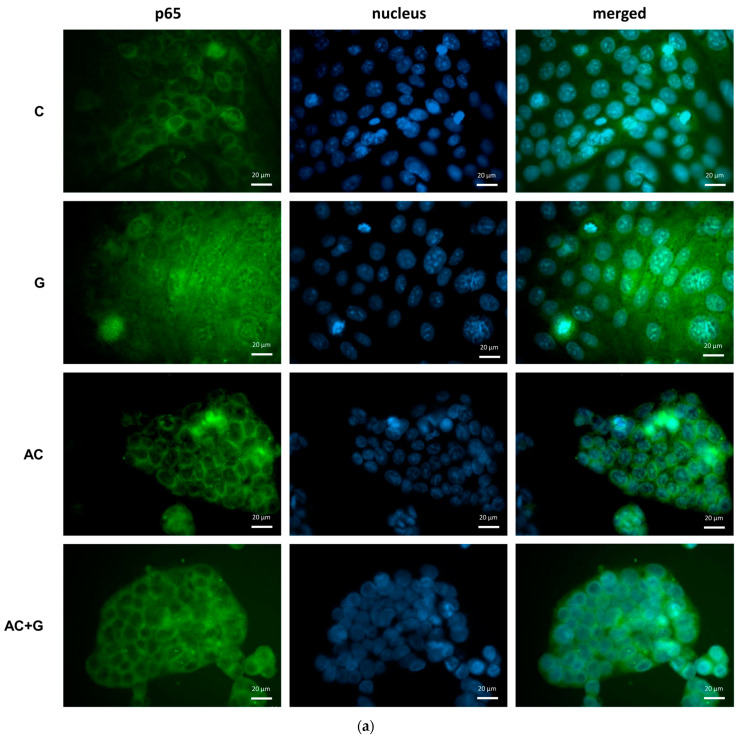

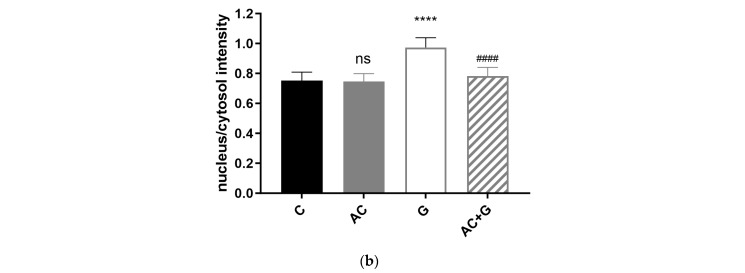
NF-κB pathway activation via p65 nuclear translocation was visualized by immunofluorescence staining in Caco-2 cells. Fluorescence microscopic images of untreated (C) or PT-gliadin-treated (G), AC-treated (AC), and in combination treated with AC and PT-gliadin (AC + G) cells are shown (**a**). Specific p65 staining is green; cell nuclei are blue. The scale bar is 20 µm. Analysis of immunofluorescent staining (**b**). Data are presented as means ± SDs, *n* = 10. **** (G vs. C) *p* < 0.0001, #### (AC + G vs. G) *p* < 0.0001. ns means not significant.

In this experiment, the effect of gliadin on the NF-κB inflammatory pathway was investigated after a short-term (60-min) treatment. The NF-κB p65 subunit was labeled with an Alexa Fluor-488-conjugated anti-p65 antibody. Activation of the NF-κB pathway by gliadin was visualized by fluorescence microscopy, as presented in Figure 6. As a result of 1 mg/mL gliadin treatment, the p65 subunit was translocated from the cytoplasm to the nucleus, so gliadin activated the inflammatory pathway compared to control cells. As a result of the AC treatment, activation did not occur, which was indicated by staining similar to the control. However, as a result of the combined treatment, the nucleus/cytosol staining intensity ratio decreased compared to the gliadin-treated group, approaching the level of the control group.

### 3.6. Cytokine Levels

**Figure 7:**
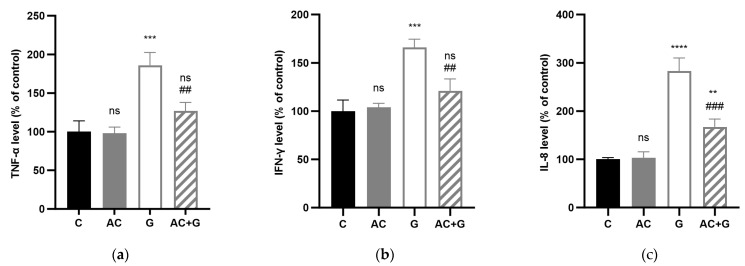
Apical TNF-α (**a**), IFN-γ (**b**), and IL-8 (**c**) levels in % of control after AC, PT-gliadin (G), and combined AC and PT-gliadin (AC + G) treatment. The control group (C) received culture medium. The monolayers were treated with 1 mg/mL PT-gliadin and/or 100 μM AC. Results are presented as means ± SDs, *n* = 3–5, **** *p* < 0.0001, *** *p* < 0.001, ** *p* < 0.01 compared to the control; ## *p* < 0.01, ### *p* < 0.001 compared to the PT-gliadin-treated group (G). The ns means non-significant compared to the untreated control group (C).

Treatment of Caco-2 cell monolayers with 1 mg/mL PT-gliadin caused significant TNF-α, IFN-γ, and IL-8 release (*p* < 0.001) in the apical compartments after 24 h of induction (see Figure 7). There was an increase in TNF-α levels of 186.22 ± 9.50%, in IFN-γ levels of 166.85 ± 8.45%, and in IL-8 production of 283.12 ± 15.64% compared to the control. The AC treatment alone did not result in significant cytokine release (98.04 ± 4.58% TNF-α, 104.13 ± 4.08% IFN-γ, and 104.09 ± 7.32% IL-8) compared to the untreated control group. However, during the combination treatment, we experienced a significantly reduced cytokine release compared to the PT-gliadin treatment, but it did not decrease to control levels. Combination treatment of AC and PT-gliadin resulted in a 127.31 ± 6.28% change in TNF-α levels, a 121.20 ± 12.34% change in IFN-γ levels, and a 167.15 ± 9.46% change in IL-8 production compared to the control.

### 3.7. Permeability Study of Small Molecular Markers

**Figure 8:**
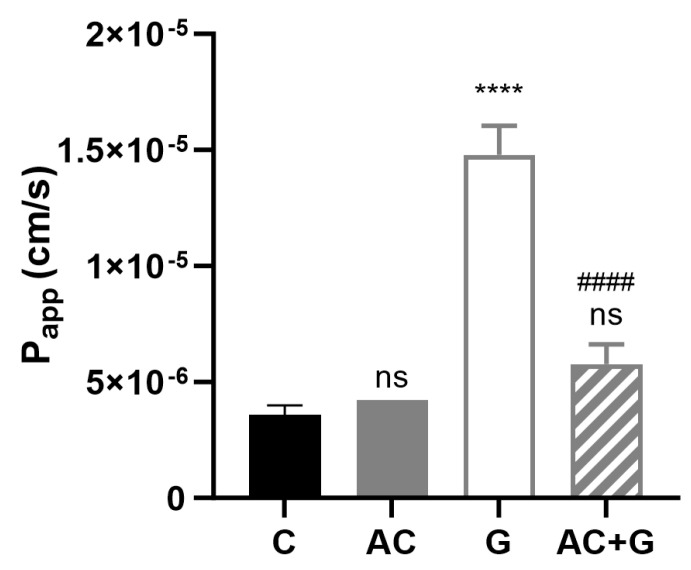
Permeability of Lucifer Yellow on confluent Caco-2 monolayer in untreated (C), PT-gliadin (G), AC-treated (AC), and in combination treated with AC and PT-gliadin (AC + G). Data are presented as means ± SDs, *n* = 3. Differences were considered significant at *p* < 0.05; **** (C vs. G) *p* < 0.0001, #### (G vs. AC + G) *p* < 0.0001; ns mean non-significant compared to the untreated group (C).

The permeability coefficient of Lucifer Yellow, as a small paracellular marker, was determined for the characterization of the permeability of the PT-gliadin-induced Caco-2 monolayer. The examination was performed from the apical to the basal side, and the P_app_ was calculated according to Equation (1), with results shown in Figure 8. The Caco-2 cell layer permeability significantly increased after PT-gliadin induction from 3.59 × 10^−6^ to 1.48 × 10^−5^ cm/s (*p* < 0.001). The AC treatment did not cause a significant difference (4.23 × 10^−6^ cm/s; *p* < 0.05) in the permeability of the cell layer compared to the control, but the AC + PT-gliadin combined treatment showed a significant improvement compared to the group of PT-gliadin treated (from 1.48 × 10^−5^ cm/s to 5.77× 10^−6^ cm/s; *p* < 0.001). Representative flow cytometric histograms of MitoSOX staining are shown in Figure S2.

### 3.8. Investigation of Mitochondrial ROS

**Figure 9:**
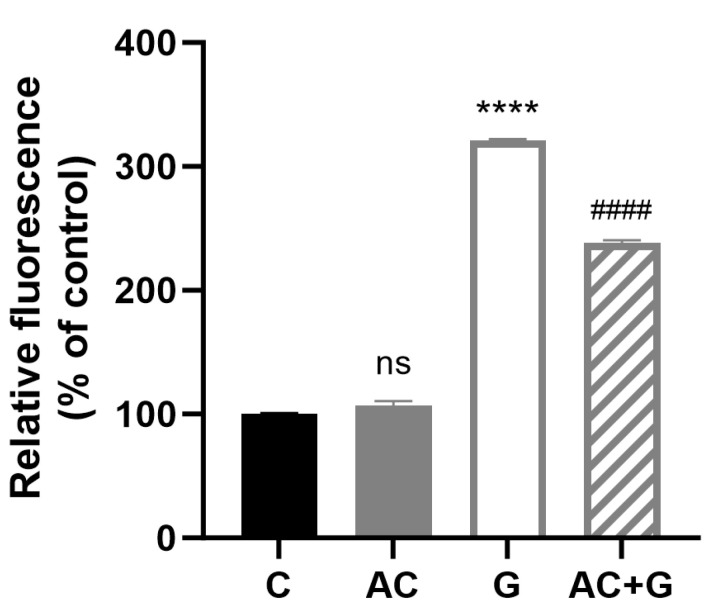
ROS production was measured by MitoSOX staining. The results were expressed in percentages compared to the control fluorescence intensity. Data are presented as means ± SDs, *n* = 3. Differences were considered significant at *p* < 0.05; **** *p* < 0.001 compared to control; **####** (G vs. AC + G) *p* < 0.01, ns mean non-significant compared to the untreated group (C).

An increase in the level of relative fluorescence was experienced (Figure 9) for the treatment of PT-gliadin, which refers to the elevated mitochondrial reactive oxygen radicals (320.96 ± 2.37%) compared to the control group. The combined treatment resulted in a significant ROS decrease compared to the PT-gliadin treatment (238.60 ± 3.31%). The AC treatment did not show a significant ROS production difference compared to the control (106.74 ± 6.38%).

## 4. Discussion

Gliadin is a protein that contains large amounts of proline and glutamine, which are incompletely digested by digestive enzymes in the human body. As a result, undigested gliadin forms several peptide fragments, such as pepsin-trypsin-resistant gliadin (PT-gliadin), in the lumen of the small intestine, which causes the release of zonulin. Zonulin is an endogenous tight junction inhibitor that reversibly regulates intestinal permeability by disrupting intercellular connections. The increased permeability of the intestinal epithelium allows paracellular transport of PT-gliadin at the brush border in addition to transcellular transport, resulting in an influx of undigested gliadins into the lamina propria [2,25]. The application of PT-gliadin permeability enhancer was chosen in this study based on previous observations.

In the development of the PT-gliadin-induced model, the first step was to determine the effective PT-gliadin concentration. Other research groups used 1 mg/mL concentrations of PT-gliadin for the induction of Caco-2 cultures [26,27,28]. Before our investigations, we performed a proliferation test on an RTCA device with a treatment of 0.1–10 mg/mL PT-gliadin. The most effective concentration was 1 mg/mL PT-gliadin because the modulating effect of 100 μM AC on the cell index was the most convincing here. This is confirmed by the results of the statistical evaluation. For further inductions, this gliadin concentration was applied.

The molecular interaction between gliadin and anthocyanin has already been investigated in some publications, and the binding of anthocyanins with different structures to gliadin has been confirmed by UV-VIS, Raman, IR, and NMR spectroscopic studies [22,23,24]. The tests were carried out at an acidic pH of 2.5, 3.0, and 4.3, a chemical reaction close to the physiological environment of the stomach. Due to the acidic environment, anthocyanidins are present in the form of a positively charged flavylium cation. When the pH increases, the flavylium cation turns into a neutral (pH 6–7) and then an anionic (pH 7–8) quinonoid base structure [29]. Examining the pH dependence of anthocyanin binding, we found that the neutral and anionic structures present at nearly neutral pH bind better to gliadin in the case of the cyanidin glycoside-rich AC we examined. The interaction showed concentration dependence in the neutral medium, but in the acidic medium, we observed an average binding of 10%. In our cell culture experiments carried out in a neutral medium, we experienced the expected physiological effects based on our previous results, which indicate the presence of an excess of the components in the AC extract. However, we confirmed that the free anthocyanin content is pH-dependent, in addition to the use of gliadin.

In enterocytes, gliadin binds to the CXCR3 receptor and triggers zonulin excretion, which results in the opening of connections between cells through signal transduction processes, which ultimately results in an increase in permeability [11,12,14]. The change in permeability and the effect on intercellular connections were investigated by TEER measurement and immunohistochemical staining of TJ proteins. The TEER change caused by gliadin, gliadin toxic peptide 31–55, and gliadin non-toxic peptide 22–39 and their time dependence were investigated by Clemente et al., and in the case of the aforementioned two materials, a decrease in TEER was experienced [14]. In our experiments, the TEER decrease after 3 days of chronic treatment was around 15%, which shows that the TEER value was stabilized on the Caco-2 monolayer. The anthocyanin treatment was also able to prevent the decrease in resistance caused by gliadin for all three days.

Zonulin plays a crucial role in modulating signaling pathways through the phosphorylation of ZO proteins, and it is a key modulator of interactions between ZO-1/2 and Occludin proteins [30]. Occludin is the first identified TJ-specific protein that contains many transmembrane domains and binds to the actin cytoskeleton through ZO-1 [31,32]. The localization of Occludin and ZO-1 was investigated on Caco-2 cultures treated with gliadin. The effect of PT-gliadin zonulin induction can be clearly illustrated by immunohistochemical staining when the zigzag-like rearrangement of intercellular connections takes place. At the same time, the effectiveness of the treatment with anthocyanin was confirmed, and TEER normalization is supported by the rearrangement visible in AC + G-treated samples. In our previous studies, which were also performed with the AC extract but under TNF-α induction, we experienced a similar staining pattern [20]. Other research groups that investigated the protective effect of polyphenols under the influence of different inflammatory inducers also confirmed the inhibition of ZO-1 redistribution [33].

The TJ and TEER investigations presented above can be considered predictive for characterizing Caco-2 monolayer permeability. The TJ rearrangement and the decrease in the resistance of the monolayer to the chronic PT-gliadin treatment are the conditions for the increase in permeability. In our experiments, PT-gliadin treatment increased permeability as expected. However, in this study, we wanted to find out whether AC would be able to moderate this increase in permeability despite the PT-gliadin-AC interaction. Our results confirmed that at the tested concentration, AC significantly improved the permeability increase induced by PT-gliadin in the case of a small-molecule paracellular transport marker. The significance of this experience lies in the fact that using cyanidin-type anthocyanins as nutrients presumably induces a favorable change in the structure of the intestinal epithelium, apart from the immunological processes taking place in the background.

The NF-κB activation pathway is a pro-inflammatory signaling pathway. NF-κB is a comprehensive regulator of cell and viral genes and consists of two subunits, p65 and p50. In normal conditions, they can be found in the cytoplasm in an inactive form, but in certain circumstances, such as inflammatory signals, the p65 subunit is translocated into the nucleus, opening the epithelial barrier at the level of signaling pathways that regulate many biological processes [34,35]. Activation is indirectly achieved by various agents; e.g., it is also triggered by the toxins of pathogenic microorganisms and gliadin [36,37]. In our previous studies, TNF-α and IL-1β were used for direct induction; however, in our current studies, Figure 4 shows that NF-κB was also induced by PT-gliadin. The full mechanism behind NF-κB activation by PT-gliadin is not completely revealed, but it is certain that PT-gliadin-induced zonulin release is MYD88-dependent. MYD88 tissue factor induces p65 translocation by TLR receptors [12]. TLR activation induces cytokine release, which is confirmed by our TNF-α, IFN-γ, and IL-8 results [38]. Anthocyanin treatment significantly reduced the translocation of p65 in our experiments, but alone, AC treatment did not result in any alteration in NF-κB activation (Figure 4b). Selective inhibition of the NF-κB pathway in Caco-2 cells confirmed that this is the main mechanism by which cyanidin-3-O-glucoside (C3G) exerts its anti-inflammatory effects [39].

Elevated ROS indicates disruption of the cell’s homeostasis, which can be compensated to some extent by the cell’s antioxidant enzyme system. However, its excessive increase initiates pathological processes. Our studies only focused on whether AC results in a change in ROS level, despite the PT-gliadin-AC interaction, in response to a change in the ROS level due to gliadin. With the endocytic uptake of smaller units of α-gliadin (P31–43), an increase in the level of free radicals was observed [40]. During the MitoSOX study, we observed a significant increase in ROS after gliadin treatment, and AC treatment did not completely protect it but significantly reduced the ROS level during the 24-h treatment. Our results are consistent with the PT-gliadin-induced ROS increase observed by Lim et al. [41]. However, in the case of anthocyanins, no studies were conducted in which the increase in ROS level induced by PT-gliadin was modulated by anthocyanins.

The decrease in ROS level achieved with the anthocyanin extract (AC + G treatment group) should also be highlighted from another point of view. Several studies have confirmed the interaction of anthocyanins with gliadin, which is most likely realized through H-bridge bonds and hydrophobic interactions. Anthocyanins adopt a quinoidal structure during the formation of the interaction, in which the A ring is most likely important [22]. However, the antioxidant capacity can mostly be attributed to the ortho-dihydroxy structure present on the B ring, which provides strong antioxidant activity in the case of the cyanidin glycosides in our extract [42].

Thanks to the interaction, anthocyanin can compensate for the immunoreactivity of gliadin. B-turns and disulfide bonds are responsible for immunoreactivity as antibody-binding epitopes. The interaction of these structural elements with anthocyanins can potentially reduce allergen immunoreactivity [23]. On the other hand, the “bound” anthocyanin retains its antioxidant capacity due to the free B ring, resulting in a reduction of ROS.

## 5. Conclusions

In several studies, Caco-2 cells were used as an in vitro model of CD intestinal epithelia for initial testing of CD treatment options. Currently, there is no available pharmacological therapy for CD patients. The only available therapy for these patients is to avoid the ingestion of gluten via a gluten-free diet. In the case of NCGS, a non-specific immune response develops, resulting in symptoms similar to CD.

As a result of gliadin treatment, oxidative damage in Caco-2 monoculture changes cell morphology, cell proliferation, and induces apoptosis. Under physiological conditions, the immune cells of the enteric system further increase these changes by secreting pro-inflammatory cytokines. That is why it is important to know, in addition to the gluten-free diet, which nutrients can have a supportive effect or be taken as dietary supplements. In this study, we proved that the anthocyanin-rich extract from sour cherries can fulfill this role, and its use in the diet as a natural source can be justified.

## Data Availability

The data presented in this study are available on request from the corresponding author.

## Supplementary Materials

The following supporting information can be downloaded at: https://www.mdpi.com/article/10.3390/nu15184022/s1, Figure S1: The percentage of viable Caco-2 cells under PT-gliadin treatment; Figure S2: Representative flow cytometric histograms of MitoSOX staining.
