# Supplementary material for: Effectiveness of Anthocyanin-Rich Sour Cherry Extract on Gliadin-Induced Caco-2 Barrier Damage
Source: Nutrients. 2023 Sep 17;15(18):4022. doi: 10.3390/nu15184022 (PMC10535085; doi:10.3390/nu15184022)
Supplement: Supplementary file 1 [file nutrients-15-04022-s001.zip › nutrients-2589766-supplementary.pdf]

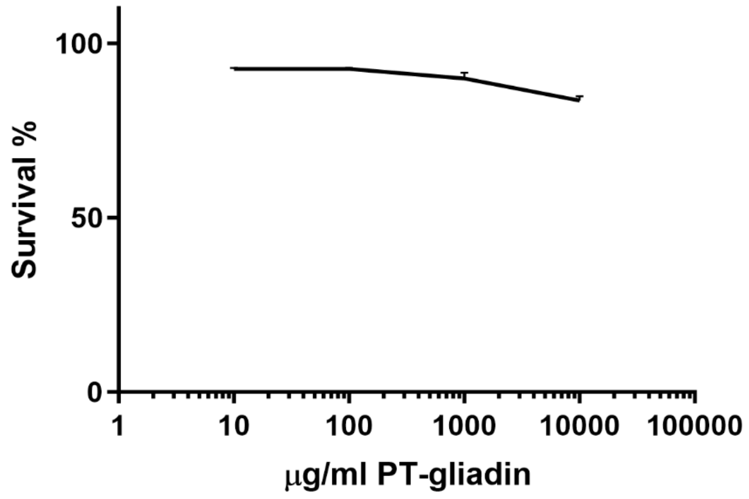

Figure S1: The percentage of viable Caco-2 cells under PT-gliadin treatment. After the treatment of Caco-2 cells with different concentrations of PT-gliadin, cells were trypsinized. Then propidium iodide (PI) was added to the cell suspension at 1 μg/ml final concentration. The flow cytometry investigation was performed immediately. The PI-negative viable cells were gated on the dot plot and expressed in percentage

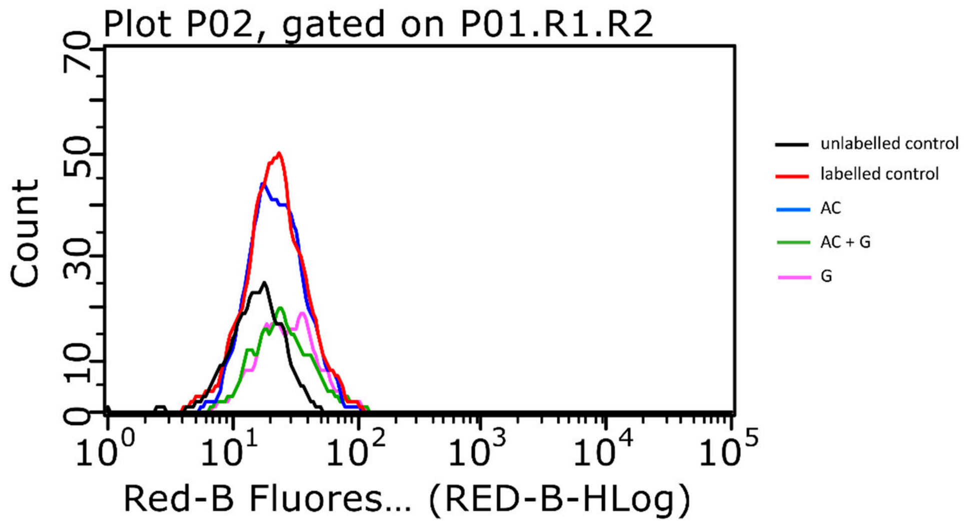

Figure S2: Representative flow cytometric histograms of MitoSOX staining. As described in Methods 2.10 cells were stained with MitoSOX red and the fluorescence intensity of the samples was measured by flow cytometry. Representative histograms of the samples are shown on the figure.
